# In situ bioremediation of Fenton’s reaction–treated oil spill site, with a soil inoculum, slow release additives, and methyl-β-cyclodextrin

**DOI:** 10.1007/s11356-020-11910-w

**Published:** 2021-01-06

**Authors:** Harri Talvenmäki, Niina Saartama, Anna Haukka, Katri Lepikkö, Virpi Pajunen, Milla Punkari, Guoyong Yan, Aki Sinkkonen, Tuomas Piepponen, Hannu Silvennoinen, Martin Romantschuk

**Affiliations:** 1 Faculty of Biological and Environmental Sciences, University of Helsinki, Niemenkatu 73, 15140 Lahti, Finland; 2 Nordic Envicon Oy, Huopalahdentie 24, 00350 Helsinki, Finland; 3 Faculty of Biological and Environmental Sciences, University of Helsinki, Viikinkaari 1, P.O. BOX 65, 00014 Helsinki, Finland; 4 Department of Geosciences and Geography, University of Helsinki, P.O. BOX 64, 00014 Helsinki, Finland; 5 MetropoliLab Oy, Viikinkaari 4, 00790 Helsinki, Finland; 6 Luke Natural Resources Institute Finland, Itäinen Pitkäkatu 4 A, 20520 Turku, Finland

**Keywords:** In situ, Bioaugmentation, Surfactant, Methyl-β-cyclodextrin, Bioremediation

## Abstract

A residential lot impacted by spills from a leaking light heating oil tank was treated with a combination of chemical oxidation and bioremediation to avoid technically challenging excavation. The tank left emptied in the ground was used for slow infiltration of the remediation additives to the low permeability, clayey soil. First, hydrogen peroxide and citrate chelate was added for Fenton’s reaction–based chemical oxidation, resulting in a ca. 50% reduction from the initial 25,000 mg/kg average oil concentration in the soil below the tank. Part of this was likely achieved through mobilization of oily soil into the tank, which was beneficial in regards to the following biological treatment. By first adding live bacteria in a soil inoculum, and then oxygen and nutrients in different forms, an approximately 90% average reduction was achieved. To further enhance the effect, methyl-β-cyclodextrin surfactant (CD) was added, resulting finally in a 98% reduction from the initial average level. The applicability of the surfactant was based on laboratory-scale tests demonstrating that CD promoted oil degradation and, unlike pine soap, was not utilized by the bacteria as a carbon source, and thus inhibiting degradation of oils regardless of the positive effect on biological activity. The effect of CD on water solubility for different hydrocarbon fractions was tested to serve as the basis for risk assessment requirements for authorizing the use of the surfactant at the site.

## Introduction

According to the Finnish Oil Pollution Fund, there are 300,000 heating oil tanks in Finland (Soini 2014). Incidents of spills reported to regional authorities annually count up to hundreds, and may involve a significant portion of individual sites regionally. All in all, these low-profile occurrences amount to the biggest environmental concern in most parts of Finland. In rare cases, these spills are associated with specific accidents, but more commonly result from normal wear and tear of the tank jackets (Puolanne et al. 1994). In these cases, it may take time before the problem becomes noticeable, and large volumes of soil can already have been affected. Excavation of tanks and the contaminated soil tend to be the standard emergency protocol whereas this may be made difficult by buildings or associated underground structures (Dahl et al. 2013). Approximately two thirds of the existing oil tanks are for residential use only (Soini 2014).

Due to the low threshold values for contamination of soil in residential areas, alternative remediation methods are rarely being considered (Sorvari et al. 2009; Reinikainen 2007). It is, however, acknowledged that the current protocol needs to be questioned in the near to immediate future: In Finland, the landfill policies concerning contaminated soil masses are becoming increasingly restrictive (MOTE 2015). Also, globally, the push towards sustainable land use and transportation require precautionary principles associated with *in situ* remediation to be re-considered (Smith 2019).

In Finland, natural attenuation is rarely enough for sufficient oil removal (Penttinen 2001). The problem with authorization of active in situ methods is that sites with lowest risks are the ones where low permeability of the soil reduces the possibility of contaminant mobilization. In these particular cases, injection of remediation additives may be difficult and low bioavailability of the contaminant prove to be the primary bottleneck for biological degradation (Martins et al. 2009). Chemical in situ treatment is similarly negatively affected by the extent to which the contaminant occurs as non-aqueous phase liquid or is absorbed into the soil matrix (Wang et al. 2013).

The most used chemical oxidant is hydrogen peroxide. In Fenton’s reaction, its breakdown is catalyzed by iron or other transition metals. The resulting hydroxyl radicals start a chain reaction with a capacity to degrade organic compounds such as hydrocarbons (Neyens and Baeyens 2003; Petri et al. 2011). This is dependent on solubility of the catalyst in acidic pH, whereas at higher pH the amount of the catalyst in soluble form can be secured by adding chelating agents (Pham 2012; Kwan and Voelker 2003; Jho et al. 2012; Sun and Pignatello 1992; Venny et al. 2012; Vicente et al. 2011).

The efficiency of Fenton-based chemical oxidation may be compromised by radical reaction inhibition or scavenging by the organic material (Lindsey and Tarr 1999; Miller and Valentine 1999; Pham 2012) or low availability of the contaminant in NAPLs or adsorbed in to soil particles, especially in clayey soils (Sellers 1999; Villa et al. 2010). Even if chemical oxidation is the target mechanism for contaminant removal, a secondary biological mechanism can contribute to the overall efficiency by increasing oxygen availability and contaminant bioavailability, especially if chemical mineralization itself has been insufficient (Talvenmäki et al. 2019; Simpanen et al. 2016; Goi et al. 2006).

In these cases, the balance between different reaction mechanisms may be difficult to define, whereas the role of biodegradation is likely to increase over time. This secondary mechanism may, however, have been impeded by the toxic effects of high H_2_O_2_, oxygen or radical concentrations, so the degraders themselves may need to be reintroduced (Tarasov et al. 2004; Büyüksönmez et al. 1998). Bioaugmentation performed with pure cultures is often reported to be unsuccessful. This is generally due to competition for limited resources between the imported strains and the native bacteria in undisturbed rather than chemically treated soil conditions (Gentry et al. 2004; Thomassin-Lacroix et al. 2002: Kauppi et al. 2011). Better results have been achieved when the natural microbial community of a particular environment is transferred in its entirety in a soil inoculum. Adaptation of the community is not required if the specific enzymatic pathway can be distributed within the indigenous population horizontally (Sarand et al. 2000). By utilizing treated soil with a similar contamination history as the base, positive results have been achieved for a multitude of organic contaminants, such as herbicides, PAHs, and diesel (Koivula et al. 2004; Kauppi et al. 2012; Sinkkonen et al. 2013).

Availability issues concerning both chemical and biological treatments can be circumvented by introducing surfactants (Chaillan et al. 2006; Martins et al. 2009). Surfactants help to overcome surface charge through changes in the interface between chemical substances of differing polarity (Khalladi et al. 2009). The additive dose should be selected according to whether the contaminant is to be flushed out physically or merely released into the aqueous phase for degradation. This is especially important for biological treatment, as mobilization may outpace bacterial digestion (Simpanen et al. 2016). When selecting the surfactant, biodegradable alternatives are preferable for environmental reasons, whereas it should be noted that highly degradable additives can also interfere with the remediation by acting as a more readily available carbon source than the contaminant itself or by having a reverse effect on bioavailability (Wang et al. 2011; Sen et al. 2012).

In laboratory-scale experiments, the effectiveness of two biodegradable surfactants, pine soap and methyl-β-cyclodextrin, on both hydrocarbon removal and biological activity was studied. Pine soap is made from pine oil, with various resin acids (mainly abietic acid and its isomers) and fatty acids (oleic and linoleic acid) acting as the surface activity–enhancing agents (Biermann 1993; Riistama et al. 2003). Pine soap is inexpensive, easily accessible in small doses, and its negative environmental impacts are considered low. Cyclodextrins are oligosaccharides produced enzymatically from starch containing substances and are able to form guest host–type complexes with many compounds in various chemical states. The hydrophobic inner centrum attaches hydrophobic compounds with van der Waals interaction, and due to the hydrophobic outer rim of the toroid shape the compound becomes water soluble (Del Valle 2004). The effect of methyl-β-cyclodextrin, the surfactant chosen on the basis of a laboratory test for the site treatment, on the water solubility of the different aliphatic and aromatic oil fractions was further examined to direct decisions at the site.

The goal of this research was to study the potential benefits of a combination treatment with Fenton’s-based chemical oxidation and a subsequent bioremediation step in situ, with emphasis on how the joint effect could be optimized by targeting the probable bottlenecks, namely the low survival of native bacteria and the low bioavailability of oil. The chosen site exemplifies both the greatest challenges involved with in situ techniques as well as the highest potential rewards: low water permeability of soil hampering infiltration but also lowering the risk of contaminant mobilization, and with only a small hotspot of contamination appearing in a vulnerable location near built structures making excavation a non-preferable procedure. The different approaches were used in combination due to following challenges encountered at the site: (1) Chemical oxidation, regardless of the initial positive results, was not considered an effective way to lower the local high hydrocarbon concentration to the Finnish guideline value levels. (2) Mechanisms leading to the heightened bioavailablity of the contaminant were observed during the chemical treatment, whereas based on the oxidant doses, biological conditions at the site were suggested to have been negatively affected. (3) Conventional biostimulation with oxygen and nutrient addition was ineffective after a certain point, and based on the soil type, low bioavailability was known to be a factor.

## Materials and methods

### Site characteristics

The study site is located in the Turenki municipality in Finland (Coordinates 60° 55′ N, 024° 38′ E) in a residential area on the premises of a single household. The site is not located within or near a major ground water area and there is no subsurface draining connecting it to neighboring properties. The average air temperature during the 30-month treatment was ca. 5 °C, with eleven of the months below 0 °C and seven above 15 °C.

**Fig. 1:**
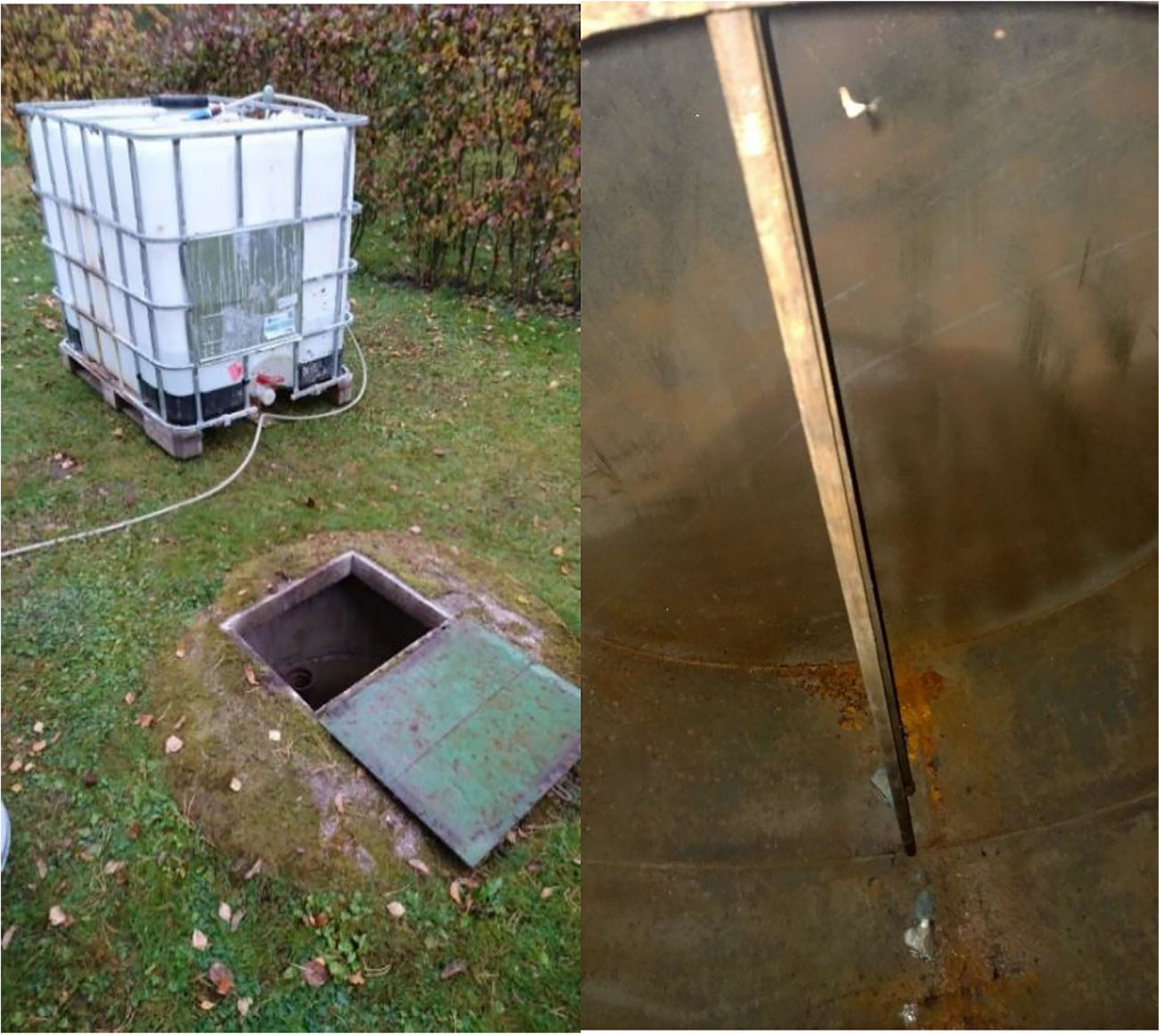
The study site. above surface view (**a**) and the tank in the original condition (**b**)

Preliminary investigations of the site were performed during 2013–2014 by the independent consultant Ramboll Finland Oy. The contamination was found to be the result of oil leakage from a faulty underground heating oil tank with one or more small holes in the tank jacket. The tank is situated approximately 1 m from the north corner of the house, with its bottom at a depth of 2.2–2.5 m. The tank has been cleaned and left intact and its removal has been ruled out due to the vulnerable location near the corner of the house (Fig. 1a and b). The contamination is unevenly distributed but generally residing at the approximate depth of the source, 2.5+ m depth. The permeable soil extends down to 2.8–3.0 m. The soil type in this zone is approximately 60% silt (< 0.063 mm) and 40% sand (0.063–2 mm).

In the preliminary investigations in 2013 by the contractor, C10–C40 concentrations were found to average 1900 mg/kg and vary between < 50 mg/kg dw to 3300 mg/kg dw in the immediate vicinity of the tank. The relative portion of the midrange (C10–C21) fractions in the total hydrocarbon concentrations varied between 66 and 90% suggesting differences in natural attenuation levels between sample points (Kostka et al. 2011; Prince 2010; Rodriguez-Blanco et al. 2010). At this point, no samples were taken directly under the tank. The soil around the tank was found highly impermeable. Approximately 10 L of water could be injected into the soil via a single vertical ground water tube (⌀ 8 cm) within a period of 2 h, and even then, the effect of injection could not be verified from a neighboring receiving tube located 20 cm away.

During the following year, 800 L of ammonium–nitrate–amended water was injected into the soil via five similar tubes during a 3-month period, increasing the nitrogen concentration from 140 mg/kg to approximately 1300 mg/kg**.** This excessive dosage was explained by an erroneous approximation of the radius of influence for a theoretical 100:10 Corg:N addition of nitrogen. The initial phosphorus content in the soil prior to any active remediation measures was 580 mg/kg, which would suggest that biodegradation was not limited by the low availability of either nitrogen or phosphorus. As such excess dose of nitrogen is found to have reverse effects on microbial respiration and hydrocarbon degradation (Fayad and Overton 1995). Since these high local levels were caused by distribution issues, it is likely that the required level was not secured across the contaminated zone. In this initial treatment, the injection of nutrients hence failed to stimulate bioremediation, and so modified and differently targeted in situ protocol was developed.

### Outline of site treatment

After the ineffective biostimulation treatment, a combination of chemical and biological treatments was performed at the site as described here. The site was treated first with Fenton’s reaction–based chemical oxidation and the biological integrity was then restored by bioaugmentation with a soil inoculant and biostimulation with various oxygen and nutrient sources which were selected based on observed changes in the water saturation conditions. Lastly, biostimulation was enhanced using biosurfactants.

Information on the benefits and risks associated with the surfactant use was collected from two small-scale laboratory tests. In the first one, two possible biosurfactant options, pine soap and methyl-β-cyclodextrin (CD), were surveyed for their effects on oil hydrocarbon removal and biological activity. After the latter was chosen for the site treatment based on the aforementioned results, its effect on the water solubility of different oil hydrocarbon fractions was tested. When the treatment is likely to enhance mobility of the contaminant, authorization of the protocol requires risk assessment based on laboratory-scale model data. For fuels, the risks are associated with particular fractions and their chemical properties, which are recognized by risk assessment tools. In this case, we used the Soilirisk assessment tool, which has been developed by the Finnish Oil Industry Service Center for standardization of the risk assessment process (OISC 2017).

### Laboratory-scale experiments

#### Effect of surfactants pine soap and methyl-β-cyclodextrin on hydrocarbon reduction and microbial activity in soil

A preliminary comparison experiment was set up to test whether the chosen surfactants, CD and pine soap, could be expected to increase biological degradation by increasing the bioavailability of the hydrocarbons rather than through acting as alternative carbon sources themselves.

Removal of oil hydrocarbons from soil—either through biodegradation, mobilization or both—as well as biological activity as measured from soil respiration, was studied in an experiment with a recycled aqueous phase. Gravelly soil with aged diesel and engine oil contamination was thoroughly mixed after rocks larger than 1 × 1 cm^2^ had been removed with a sieve. This soil, which was coarser than the one found at the site, was chosen to regulate the filtering duration and downplay the role of the edge effect. Soil organic carbon, nitrogen, and phosphorus concentrations were analyzed. Nitrogen content was measured as being below the threshold value and thus the limiting nutrient.

**Fig. 2:**
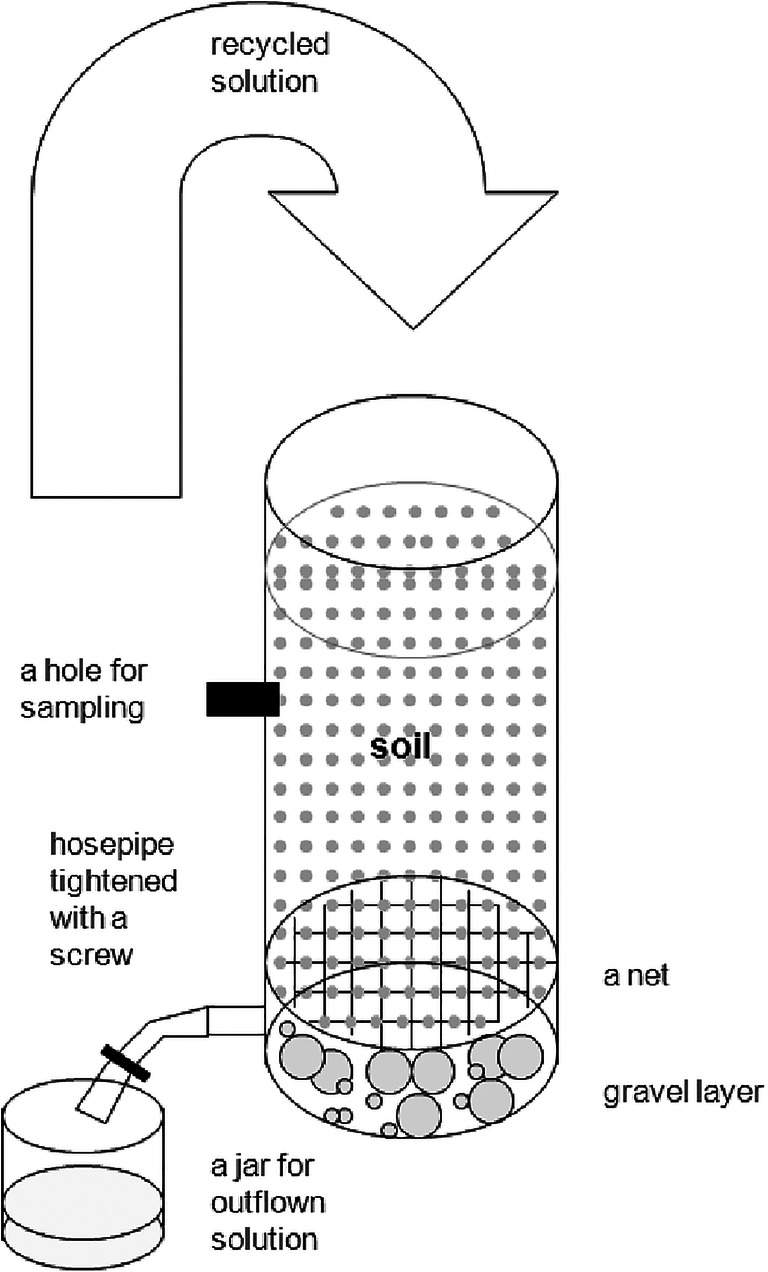
The construction of experimental cylinders. Height 50 cm, diameter 10 cm

The soil was placed in 18 plastic (⌀ 10 cm, 50 cm) tubes with 3.5 L of soil each, with the aqueous phase of 1.5 L recycled through them (Fig. 2). A thin layer of gravel and a plastic membrane was laid below the soil in the tubes to enable water movement but prevent soil leakage. A small horizontal hole was drilled in the middle of the tubes for sampling.

Either nutrients and/or surfactants were added to the recycled water phase. The experiment consisted of six different treatments, all executed as three replicates. The treatments were (i) control without any additions, (ii) liquid pine soap, (iii) liquid pine soap plus nutrients, (iv) nutrients, (v) cyclodextrin and (vi) cyclodextrin plus nutrients.

The surfactant concentration in the solutions was approximately 1%. Nitrogen was added as an ammonium nitrate mix (nitrogen dose 12 mM) and phosphorus as K_2_HPO4 + NaH_2_PO_4_ buffer in dose 710 mg/l (15 mM P). Solutions were adjusted to pH 7 by adding either base (NaOH) or acid (HCl).

The experiment lasted for 13 weeks during which the solutions were recycled trough the soil tubes 22 times. Before returning the solutions that had passed through the soil back into the tubes, pH was determined and the solutions were aerated by vigorous bubbling with aquarium pumps for 1 h. Solutions containing liquid pine soap were not aerated due to foam formation. Samples from the soil were withdrawn four times, 1 week, 3 weeks, 9 weeks, and 13 weeks after the start of the experiment, in ca. 50 g mass.

Oil hydrocarbon concentrations were measured with GC-FID as described in “Analyses” and microbial activity was measured as respiration based on CO_2_ production. CO_2_ is suggested in this case to result entirely from mineralization of the organic contaminant (Wade 2018; Santruckova 1993). While high respiration values are not always connected to increase in biomass, respiration level itself is a more direct indicator of organic matter decomposition rate (Rui et al. 2016). Respiration was measured from the total carbon in the airspace of the vial was measured with Teledyne Tekmar Apollo 9000 Combustion TOC Analyzer. For this, approximately 20 g of soil was weighed into a glass vial that was capped and left to stand for 30 min. The rate was calculated as the difference in CO_2_ concentration in the gaseous phase between measurements at 0 and 3 hours.

#### Solubilization of hydrocarbon fractions

The efficiency of CD to dissolve aliphatic and aromatic hydrocarbon fractions with the type of soil type found at the site was tested in laboratory scale using clayey soil from an alternative site, with aged heating oil contamination. In situ biostimulation had been performed at the site, suggesting that the more bioavailable fractions had already been degraded, increasing the comparability between the sites. The *C*_org_:N:P ratio of this soil was 100:3:11.

The soil was sieved (6 × 6 mm^2^) and thoroughly mixed. The oil content after the homogenization was measured from five replicates. In the experiment, 200 g of soil (dry matter content 88%) was weighed into Erlenmeyer flasks and 300 ml of solution was added. CD was tested in doses of 5% and 1% (w:v), and compared to a water control. All treatments were performed as three replicates. During the first part of the treatment, the flasks were placed in a shaker (150 rpm, 30 mm orbit) for 1-h sessions after which the phases were allowed to separate before the following run. This procedure was repeated five times in total. After the initial sampling, it was repeated in a similar manner but with shaking runs lasting for 5 h. A 250-ml water sample was withdrawn after both of the steps and a compensating treatment solution volume was added.

The results from the test were not presumed to entirely correspond with those from a study or site treatment where the solvent would be instilled or circulated. In this manner, what could be studied was how the addition of CD could enhance the mobilization of non-volatile fractions of oil hydrocarbons in optimal conditions in a thoroughly water-saturated zone. All samples were analyzed for fractions in the C10–C21, C21–C40, and C10–C40 ranges as described in “Analyses.” A complete fragment analysis was performed on a single sample per treatment.

### Site treatment

#### Sampling and injection through the tank jacket

The soil immediately below and around the tank was suspected to consist of coarser filling material, and hence act as a continuing contamination source due to the heightened permeability and proximity to the source. Because of these factors, this zone was presumed to respond more favorably to remediation efforts. Due to this higher permeability and the suspected location of the hot spot, sampling and water injections were performed through holes in the tank itself.

**Fig. 3:**
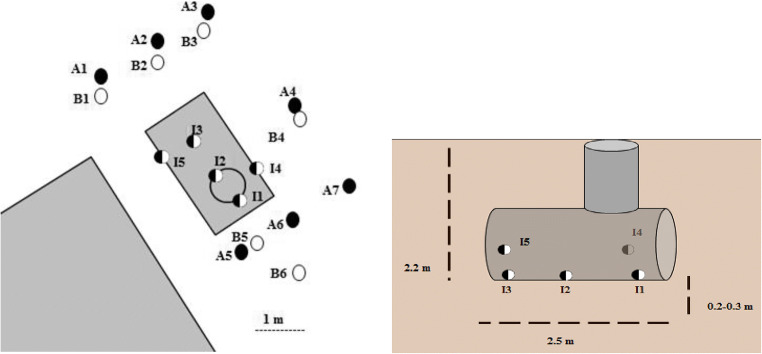
Technical drawing of the site with sampling spots marked, from above (**a**) and a vertical cut view (**b**). Black sampling spot color indicates sampling performed before remediation activities, white color indicates sampling after or during remediation

For this, five holes (⌀ 4 cm) were drilled (Fig. 3a and b). Three holes were located at the bottom axis and two holes higher up the jacket approximately 15–20 cm from the bottom. Soil samples ca. 60 g in mass were withdrawn with a ⌀ 3.5 cm plastic auger from depths 0–10 cm, 10–20 cm, and 20–30 cm, found to be the maximum depth of the penetrable layer. Higher concentrations of oil hydrocarbons were measured from the initial samples than in any previous investigations, at levels exceeding 10,000 mg/kg (Table 3). All treatments were performed by pumping injection fluids into the tank, from which the fluids slowly could infiltrate the surrounding soil through the holes. Soil mobilization during infiltration allowed subsequent samplings to be performed using the same holes.

#### Chemical oxidation (4 weeks)

Chemical oxidation required low natural organic matter content in the treated zone, low soil oxidant demand, and conditions in which the catalyst remain in a soluble form (Haselow et al. 2003; Pham 2012; Kwan and Voelker 2003; Jho et al. 2012; Sun and Pignatello 1992; Venny et al. 2012; Vicente et al. 2011 ). Non-water–saturated conditions were also required as otherwise infiltration of the peroxide may have been limited. Soil oxidant demand was tested as described in Talvenmäki et al. (2019) as surpassing 800 mg KMnO_4_ /100 g of soil even when visible organic material such as roots had been removed. However, as the majority of the contamination was suggested to appear within a small quantity of soil immediately below the tank, chemical oxidation was still considered an applicable method.

**Fig. 4.**
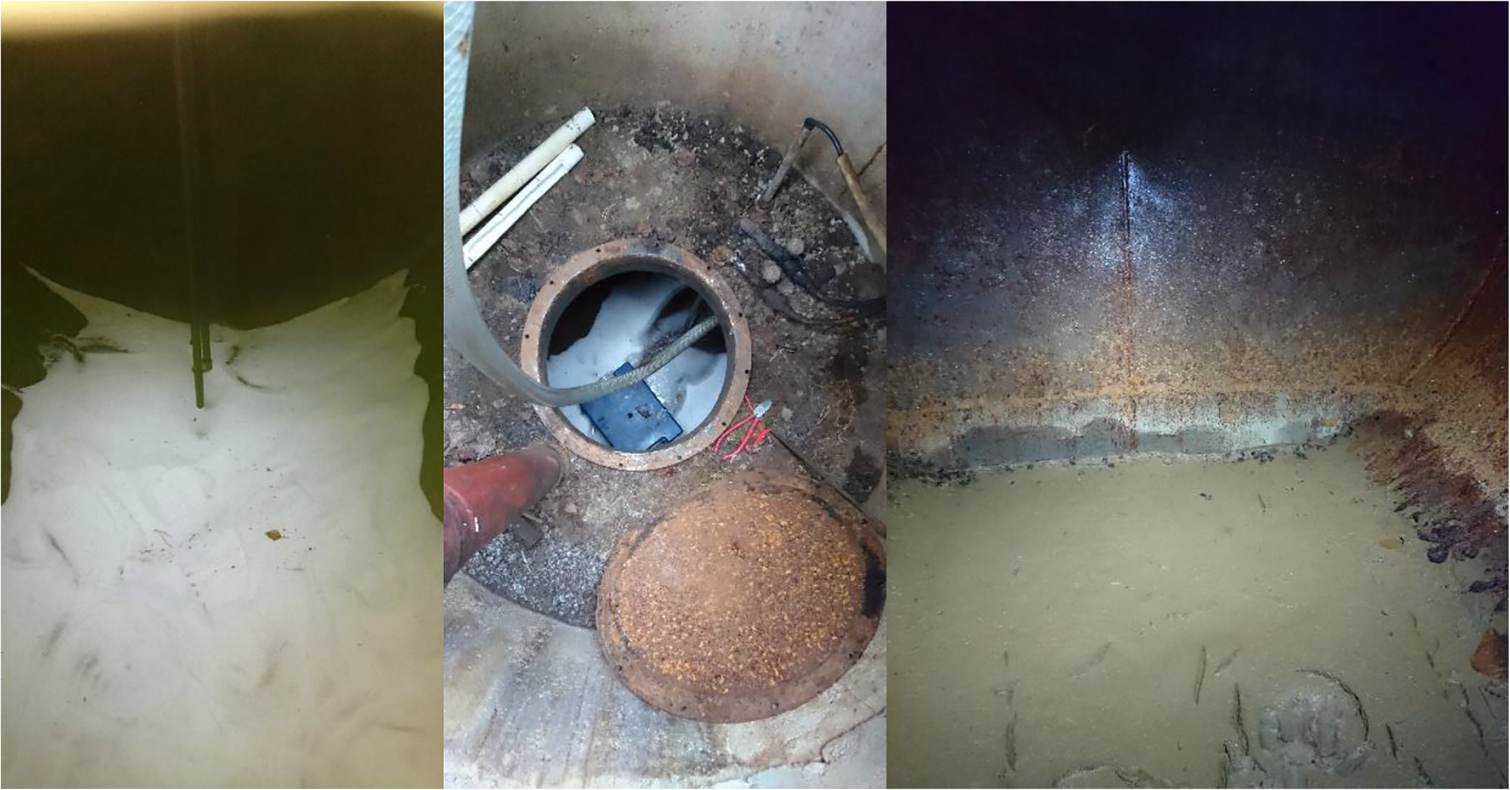
Chemical treatment. Addition of citrate (**a**), injection of peroxide (**b**), resulting soil mobilization (**c**)

The soil was initially treated with chemical oxidation based on Fenton’s reagent, catalyzed by soil minerals alone. The availability of catalyst was tested by mixing soil with H_2_O_2_. Soil pH 6.7 was found to be too high for non-chelated chemical oxidation, and so citrate chelate was used (Vicente et al. 2011; Lewis et al. 2009). Twenty-five kilograms of tri-sodium citrate was added into the tank with 0.5 m^3^ of water (Fig. 4a). One cubic meter of 25% H_2_O_2_ was then added (Fig. 4b). The injected mixture was found to infiltrate the soil within 12 to 24 h. The H_2_O_2_ injection was repeated after 2 weeks with soil sampling performed immediately before each infiltration event.

#### Bioaugmentation with soil inoculum and non-saturated soil biostimulation (10 months)

Since H_2_O_2_, the released oxygen and the radicals are all known to be toxic in high concentrations, actions were taken to introduce living bacteria following the Fenton’s treatment (Tarasov et al. 2004). A 40 kg soil inoculum, consisting of treated, aged diesel–contaminated soil originating from the published study of Liu et al. (2019), was placed into the tank. The inoculum was a 50/50 mix from a control treatment and treatment with CaO_2_ in dose 2%. In both treatments, the original oil concentration of 680–800 mg/kg had decreased by 40–61% during a period of 18 weeks (Liu et al. 2019). The suitability of the inoculum was tested by determining the taxonomic composition of the bacterial community in the two soils by sequence analysis of the 16S rRNA gene pool (described in Hui et al. 2019). The community structure was calculated using Mothur software (Appendix 1 Table 4) as described by Roslund et al. (2019) and Grönroos et al. (2019). Relative abundance of phyla and classes within Proteobacteria were plotted using R as described by Hui et al. (2019).

Nitrogen was added together with the inoculant as 10 kg of agricultural ammonium–nitrate fertilizer (26% total nitrogen, 13% ammonium nitrogen, 13% nitrate nitrogen). Additives were mixed with the soil in the tank, and introduced deeper into the contaminated zone with a 0.2 m^3^ dose of water added twice a month by the landowner. This frequency was selected to keep the soil moist but allow for sufficient periods of aerobic conditions in the soil between the additions. The soil was sampled after 2.5, 4, and 10 months.

#### Low-maintentance biostimulation with slow release compounds (6 months)

Biostimulation was continued in a modified form, with slow release fertilizers allowing for a low-maintenance treatment. A meat industry waste product, meat and bone meal, was used as a slow release source for not only nitrogen but also for phosphorus, potassium, and calcium (Liu et al. 2019). Twenty kilograms of meat and bone meal with ca. 50% protein content was mixed with the soil in the tank. H_2_O_2_ was added in low concentrations (0.5–1% of the introduced water volume) to increase the oxygen level (Goi et al. 2006; Simpanen et al. 2016). After 6 months, the soil was sampled.

#### Methyl-β-cyclodextrin enhanced ciostimulation in the saturated zone (12 months)

H_2_O_2_ was first used as the oxygen source as before. After 2 months, as the contaminated zone was now completely water-saturated, 10 kg of CaO_2_ was added to the tank to provide oxygen as in a slow release aquatic zone application (Nykänen et al. 2012). To enhance the efficiency of biostimulation by improving the bioavailability of the contaminant, 10 L of CD was added. The addition resulted in a 5% concentration when diluted of 100 L of water, which was the coarse estimate for the initial water volume in the tank. The use of a biosurfactant was justified through water-saturated conditions leading to slow migration of the aqueous phase and the assumption that bioavailable fractions had been degraded through previous biostimulation phases.

After 12 months, the soil was sampled from the monitoring holes in single 20- to 30-cm soil columns, since water and watery sludge in the tank prevented non-disturbed sampling of distinct soil layers. Oil concentration of the water in the tank was also analyzed. An independent party, Vahanen Oy consultants, performed the final investigations of the area with samples withdrawn from a wider area around the tank, corresponding with previous full area investigations in 2014 (Fig. 3a). A risk assessment was performed based on these results.

### Analyses

In each test, oil hydrocarbons C10–C40 in soil were determined with GC-FID according to standard methods ISO 16703 (soil) and 9377-2 (water) either by the research team (circulation test) or by the accredited lab Synlab Oy, Finland. The analyses connected to the broader site investigations by contractors were executed at various accredited laboratories (Synlab Oy, Eurofins, ALS Finland) with the aforementioned method, or accredited in-house methods based on similar GC-FID technology. Due to the number of laboratories involved, the protocol can be expected to vary within that described in the ISO standard. Inner standards are required in the method for validation of the protocol. The scope of the ISO 16703 standard is all aliphatic and aromatic hydrocarbons with boiling points between those of n-alkanes C10 (n-decane) to C40 (n-tetracontane) (ISO 2004). This range is further separated into midrange and heavy distillates by a C21 marker (n-henicosane). These groupings follow the Finnish threshold and guideline values for oil hydrocarbons (Ministry of Environment 2007). In the analyses performed by the research team, 2 ml of hexane-based extraction solution was used for 2 g of soil. Fraction analysis was performed by Synlab Oy with an accredited in-house method.

Nitrogen and phosphorus contents in soil were measured by Eurofins according to accredited in-house methods RA2021 and RA3000 respectively. Organic carbon content was calculated from LOI (550^o^C) with organic carbon/organic matter ratio 0.50. pH was measured according to standard method ISO 10390, or in the circulation test with a similar procedure but with ultra-pure (mQ) water added in 5:1 water to soil ratio, and with shortened shaking (5 min) and incubation (2 h) periods.

### Statistical testing

To test the effects of nutrients, pine soap and cyclodextrin—and especially the nutrients × pine soap and nutrients × cyclodextrin interaction effects—on microbial activity and diesel content in the circulation experiment, log-transformed data was analyzed using two repeated measures ANOVA: One included time, nutrients, and pine soap as explaining variables, the other time, nutrients and cyclodextrin. In the dissolution test for CD, statistical analysis on the effect of treatment on the C10–C21, C21–C40, and C10–C40 concentrations in the aqueous phase was performed with a one-way Anova, and the differences between groups validated with Student-Newman-Keuls post hoc test. The homogeneity of variances was tested with Levene’s test. For the rule to apply, results from the initial sampling were log-transformed. Normality was assumed but could not be verified due to low number of measurements

### Reagents

Havu pine soap is produced by Henkel Norden Oy. CAVASOL® W7 M TL 50% methyl-β-cyclodextrin was purchased from Wacker Fine Chemicals. H_2_O_2_ at 35% and 50% solutions was purchased from Bang & Bonsomer Group Oy in Finland and Granular 70CG CaO_2_ from Solvay GBU. Nitrogen fertilizers, urea, and Suomensalpietari (ammonium nitrate) were obtained from Yara Suomi Oy, Finland. K_2_HPO_4_ and NaH_2_PO_4_*H_2_O from Sigma-Aldrich were used for the phosphate buffer. The citrate product applied was W302600-25KG-K sodium citrate dihydrate ≥ 99 from Sigma-Aldrich.

## Results and discussion

### Laboratory-scale tests

#### Preliminary surfactant selection

**Fig. 5:**
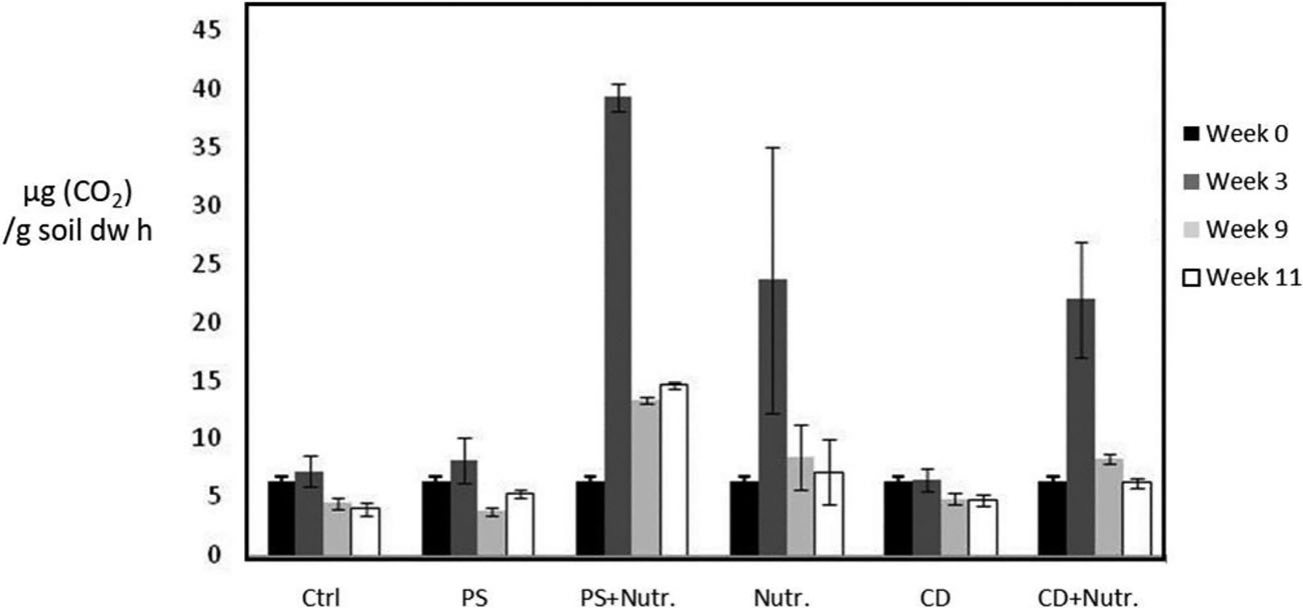
Microbial activity as μg CO_2_/g soil dw h produced over time in the different treatments. Standard deviation shown by error bars. Ctrl, control; PS, pine soap; Nutr., nutrients; CD, cyclodextrin

**Fig. 6:**
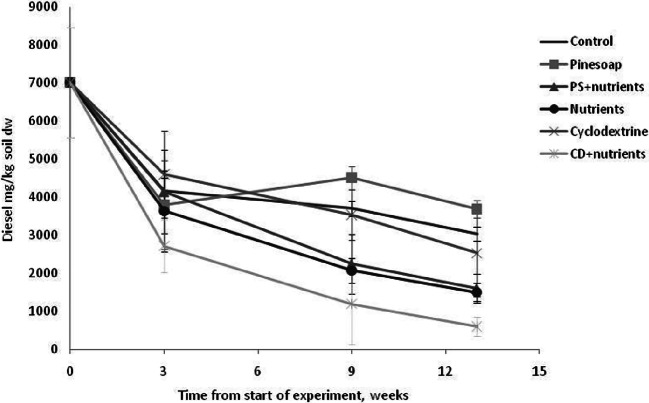
The changes in diesel concentrations (mg/kg soil dw) in different treatments during the experiment. The last oil content analysis was performed on week 13. Error bars show standard deviation

For the soil used in the circulation experiment, carbon availability was not the rate limiting factor while additional nutrient availability (N, P) apparently was. This is shown by the fact that addition of pine soap alone had no effect on microbial activity as measured by generation of CO_2_ at week 3, while nutrients alone had a significant effect on activity (Table 1, Fig. 5). The positive effect of nutrients was increased by pine soap but not by CD, indicating that the former itself may function as a carbon source. This notion is confirmed by the diesel oil degradation data as pine soap alone inhibited diesel degradation in comparison to the control treatment (Fig. 6) apparently by providing an easier source of carbon and energy and by competing for limited resources, while CD alone had no effect observable by increases in emissions compared to nutrients alone. The positive effect of nutrient addition on diesel removal was increased by CD, suggesting that improved bioavailability is a relevant factor, as shown earlier for PAHs (Simpanen et al. 2016)

**Table 1:** Between-subjects and within subjects effects in repeated measures ANOVA on microbial activity (μg CO_2_/g soil dw h) and in C10–C40 hydrocarbon concentrations (mg/kg soil dw), in mesocosms with treatments of nutrients and pine soap or cyclodextrin. The data has been LOG10 transformed

	Microbial activity			c(C10-C40)		
	F	df	*p*	*F*	df	*p*
Intercept	1497.390	1	< .0005	784.608	1	< .0005
Nutrients	50.781	1	< .0005	68.253	1	< .0005
CD	0.037	1	0.852	6.067	1	0.039
Nutrients × CD	0.303	1	0.599	4.197	1	0.075
Intercept	1652.951	1	< .0005	582.907	1	< .0005
Nutrients	97.276	1	< .0005	24.696	1	.002
PS	10.939	1	0.013	1.666	1	0.238
Nutrients × PS	5.838	1	0.046	0.096	1	0.765
Time	54.522	2	< .0005	47.000	1	< .0005
Time × nutrients	6.958	2	0.008	6.173	1	0.042
Time × PS	2.880	2	0.090	0.101	1	0.760
Time × nutrients × PS	0.404	2	0.675	0.234	1	0.643
Time	64.459	2	< .0005	42.069	1	< .0005
Time × nutrients	16.569	2	< .0005	0.813	1	0.394
Time × CD	0.131	2	0.878	0.647	1	0.448
Time × nutrients × CD	0.429	2	0.659	0.704	1	0.426

The stimulatory effect of CD could not be verified statistically, even though the highest reductions were achieved when CD treatment was coupled with nutrient addition. The balance between the two mechanisms, biological and mechanical, was not quantified. In the study by Simpanen et al. (2016), similar 1% dose of CD resulted in biological degradation of PAHs, whereas a 5% dose increased the availability of the contaminant to levels where mobilization appeared to be primary removal mechanism. Additionally to differences in compounds, this balance between mechanisms would also likely differ for other soil types, as well as for non-disturbed soil. The result could not therefore be extrapolated to field as such. The moderate effect on respiration is likely explained by carbon availability. The effect of contaminant availability is likewise factoring the degree to which the additives will be utilized as carbon sources, whereas already according to the lab scale model, pine soap could be ruled out for in situ applications for contaminated soil of any type.

When the suggested bottleneck of nutrient availability was removed, CD appeared to heighten biodegradation during the initial stages similarly to what has been observed by Molnár et al. (2005) and also lead to lower final concentrations, suggesting higher benefits from surfactant use once the readily available fractions have already been exhausted (Khalladi et al. 2009). Positive effects of CD addition on biodegradation have been noted also by Fenyvesi et al. (2008) and Taccari et al. (2012). Molnár et al. (2005) connect the initial differences to a shortened microbial lag period, but only for soils in which carbon availability is the primary bottleneck. While the peaks in nutrient and nutrient + CD treatments did not differ significantly, the latter phenomenon would require that the period of heightened activity lasts longer. Neither reduced lag periods nor lengthened high activity could be verified from the CO_2_ data.

Only minimal variation in organic matter content was observed between treatments and within the survey era. The organic matter content was between 1.3 and 1.9% during the experiment in all the treatments except week 1 in the CD treatment, when an average organic matter content of 3.2% was measured due to a single outlier value.

#### Methyl-β-cyclodextrin effect on water solubility for different oil hydrocarbon fractions

**Fig. 7:**
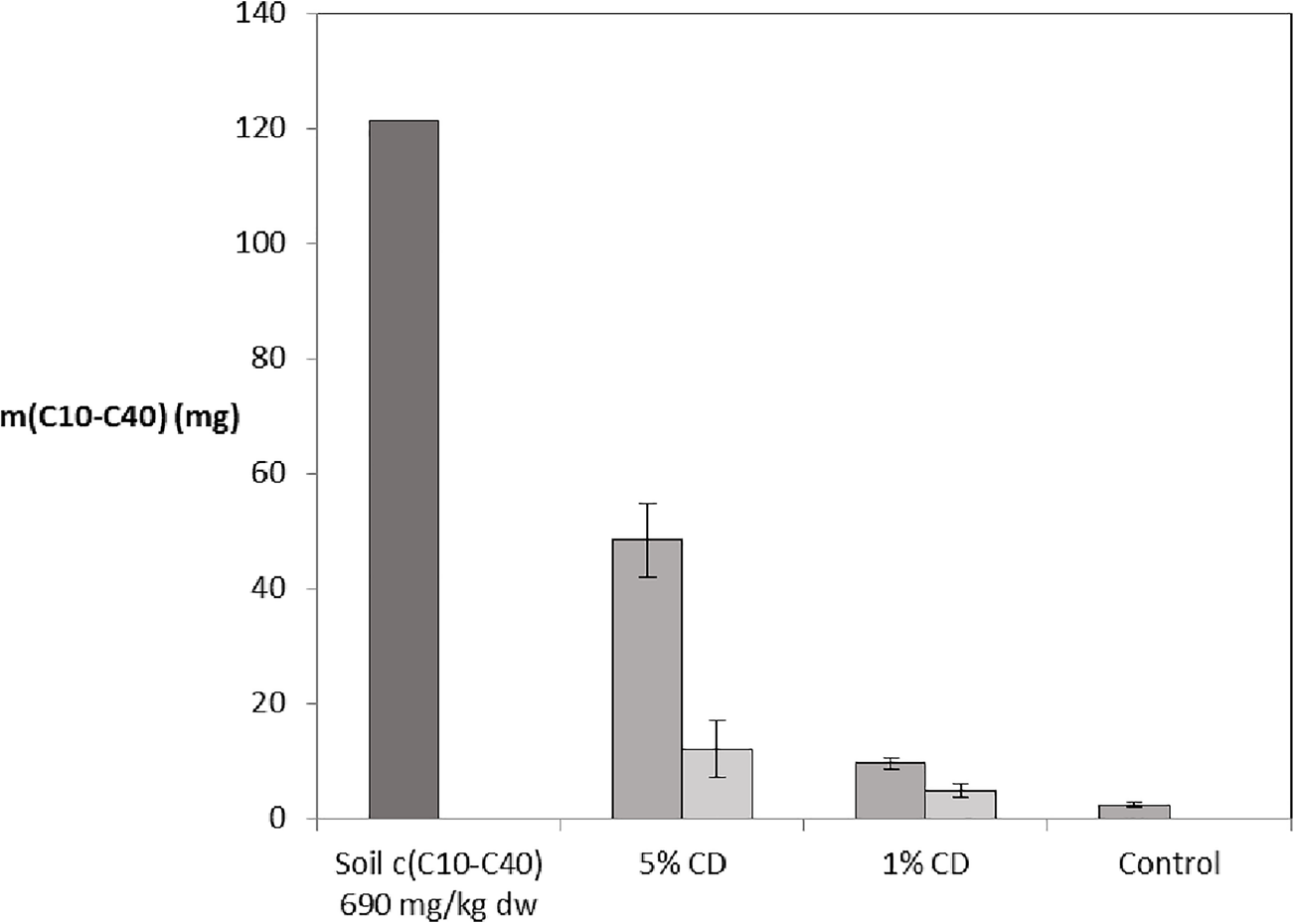
The portion of C10–C40 in soil originally dissolved into the water phase, after five 1-h periods of shaking, and after subsequent five 5-h periods of shaking. Standard deviation is shown by error bars (*n* = 3)

The concentration of CD was found to affect the solubility of oil hydrocarbons in a significant manner (*p* < 0.001), with apparent differences between all treatments (*p* < 0.01) for all fraction ranges with results from multiple replicates (C10–C21, C21–C40, and C10–C40, Fig. 7). For both the 1% and 5% CD concentrations, a similar amount of contaminant was solubilized per CD concentration during the first stage (32 mg of C10–C40/g of CD).

Similar statistical differences were observed during the subsequent test with significant results between treatments in all fraction ranges. However, an obvious decline in the treatment efficiency was observed, now only ca. 8 and 16 mg of C10–C40/g of CD for the 5% and 1% treatments respectively, in spite of the fivefold shaking time.

The test soil had been withdrawn from a site where biostimulation without surfactants had been performed for several years, and the original concentrations in soil indicated that aliphatic compounds in ranges C12–C16 and C16–C35 had been the most recalcitrant. While the solubility of all aromatic and aliphatic compounds was now affected by CD, no special value in regards to the most hydrophobic compounds was detected (Table 2). The fragment analysis suggested that the largest impact was on compounds in the aromatic C16–C21 range, despite the low initial concentration in soil.

**Table 2:** The portion of the soil bound oil hydrocarbon fractions dissolved into the water phase at the two sampling instances (w:w (%)). The results from the second sampling take into account the lowered starting level. The results come from a single sample per treatment. The total reductions in range C10–C40 are comparable with the average results from all three replicas (marked *)

	c. in soil	5% CD			1% CD			Control		
	(mg/kg dw)	1.	2.	total	1.	2.	total	1.	2.	total
Arom. C10–C12	< 30									
Arom. C12–C16	44	54%	15%	61%	10%	2%	12%	1%	0%	1%
Arom. C16–C21	30	43%	85%	91%	11%	0%	11%	0%	0%	1%
Arom.C21–C35	< 30									
Aliph. C10–C12	84	48%	− 2%	47%	6%	0%	5%	2%	0%	2%
Aliph. C12–C16	290	64%	10%	67%	12%	3%	15%	2%	0%	2%
Aliph. C16–C35	490	34%	16%	45%	7%	4%	11%	1%	0%	1%
C10–C21	790	48%	13%	55%	9%	3%	12%	1%	0%	1%
C21–C40	150	34%	17%	45%	6%	3%	8%	4%	− 1%	3%
C10–C40	950	45%	12%	52%	9%	3%	11%	2%	0%	1%
*C10–C40 average in 3 replicas		40%	18%	51%	8%	4%	12%	2%	0%	1%

The higher concentration of CD did not only enhance the initial effect, but the total effect as well, as with both 5% and 1% dose more efficient solubilization was achieved during the initial run. This means that the total mass of dissolved fractions was not strictly related to the total amount of CD molecules added. Had that been the case, the efficiency of the 1% CD treatment would not have been negatively affected until a specific threshold was being reached and similar results would be achieved by adding one larger CD dose or a number of smaller doses at different times. Injection of a single large dose therefore proved to be more cost effective whereas in terms of in situ treatments, this choice would likely affect whether or not the dissolved contaminant would be biodegraded rapidly enough so as not to increase the treatment associated risks to an undesired level (Simpanen et al. 2016). The technical grade CD product used in the experiment is only available in 200+ kg amounts, and even with the lowered purity level, it is expensive in comparison to common soaps such as pine oils. Based on this, it is still relevant to test other biosurfactants case-by-case.

### Site treatment

The initial chemical oxidation with H_2_O_2_ and citrate resulted in a decrease in average hydrocarbon concentrations under the tank jacket (Table 3). Mobilization of soil during treatment was found to transport soil from the flanks of the tank, leaving the sides hollow and therefore unfit for sampling.

**Table 3:** Results from the site treatment. * indicate results from a particular depth come from a composite sample. Missing value indicates that a sample could not be withdrawn. For below LOQ values, the LOQ value was used in the calculations. Average values per spot/per depth on gray background. Italicized values imply C10–C21 to C10–C40 ratio below 50% or values below LOQ. Final column indicates total reductions in average values per spot/depth from the initial values (%) and change from the previous sampling (± %). Results are calculated from the three bottom holes. Due to soil mobilization, the spots higher up the tank sides were left hollow and therefore unfit for sampling during the majority of the stages, and these values were excluded

Original level	*I*1	*I*2	*I*3	Average	Total reductions in average c per spot/depth (change from the previous stage)
				(per depth)	
	C10–C21/C10–C40				
0–10 cm	620/1000	20,000/26000	28,000/35000	16,000/21000	
10–20 cm	27,000/31000	38,000/47000	29,000/36000	31,000/38000	
20–30 cm	6100/7000	31,000/37000	5700/6900	14,000/17000	
Average (per spot)	11,000/13000	30,000/37000	21,000/26000	21,000/25000	
1. Chemical treatment					
0–10 cm	*< 50/54*	*< 50/86*	10,000/12000	3300/4000	
10–20 cm	9500/11000	10,000/13000	2200/2800	7200/8900	
20–30 cm	8600/9900	10,000/13000		9300/11000	
Average (per spot)	6100/7000	6700/9000	6100/7400	6500/7900	69/68%
2. chemical treatment					
0–10 cm	430/840	11,000/14000	120/210	3900/5000	
10–20 cm	4100/5100	26,000/31000	*1400/3000*	11,000/13000	
20–30 cm		19,000/23000		19,000/23000	
average (per spot)	2300/2800	19,000/23000	*760/1600*	9300/11000	56/55%
Biostimulation 2, 5 months					(− 43/− 39%)
0–10 cm	230/330	540/750	420/710	390/600	
10–20 cm	**<** *50/54*	25,000/29000	460/760	9000/9900	
20–30 cm	*170/690*	20,000/23000		10,000/12000	
Average (per spot)	*150/360*	15,000/17000	440/740	5800/6800	72/73%
Biostimulation 4 months					(38/40%)
0–10 cm	*57/150*	*56/330*	*< 50/170*	*54/210*	
10–20 cm	270/480	4400/5600	3300/4200	2700/3500	
20–30 cm	1300/1800	5300/7000		3300/4400	
Average (per spot)	540/810	3300/4300	1700/2200	1900/2600	91/90%
Biostimulation 10 months					(67/63%)
0–10 cm	*< 50/< 50*	*71/160*	*< 50/130*	*57/150*	
10–20 cm	3400/4100	580/820	730/960	1600/2000	
20–30 cm	3200/3800	2500/3300		2900/3600	
Average (per spot)	2200/2700	1100/1400	390/550	1400/1700	93/93%
Biostimulation 16 months					(26/33%)
0–10 cm	*63/140*	270/520	*75/210*	*140/290*	
10–20 cm	*60/140*	4700/5900	180/290	1600/2100	
20–30 cm	3000/3500	6000/7200		4500/5400	
Average (per spot)	1000/1300	3700/4500	130/250	1900/2300	91/91%
Biostimulation 28 months					(− 36/− 35%)
0–10 cm	*170/480*	*150/450*	*140/370*	*150/430*	
10–20 cm	170*/480*	150/450*	140*/370*	*150/430*	
20–30 cm	170*/480*	150/450*		*160/470*	
Average (per spot)	*170/480*	*150/450*	*140/370*	*150/440*	99/98%
					(92/81%)

During the reaction, water and clayey sludge was observed to be transported mainly through the tank itself, as foaming water was rising to the surface visibly through the tank manhole. Furthermore, the treatment had also driven approximately 0.5 m^3^ of soil into the tank through the sampling holes (Fig. 4c).

Contaminant reductions in ca. 1/2–2/3 range were achieved with two peroxide injections. The fact that the initial effect could not be repeated, rather on the contrary, suggests that the effect of chemical mineralization alone was limited in this particular situation. The oil hydrocarbon content of the soil in the tank was approximately 600 mg/kg dw. The permeable layer below the tank was only 0.2–0.3 m deep. Considering the exit route of the water through the porous soil, the radius of influence was likely limited to the immediate surroundings of the tank. If also the total mass of the contaminant in the soil was low, which could not be ruled out, oil concentrations under the tank were reduced primarily through soil mixing and dilution. Contaminant-to-oxidant stoichiometry could not be determined as the concentrations were known to be heterogeneously distributed and also the soil mass within the radius of impact remained unknown.

During chemical oxidation, the H_2_O_2_ concentration in the tank was 17%, and due to the low radius of influence, the concentration in the immediate zone was likely rather close to the initial level. The effect of high peroxide, oxygen, and possibly radical concentrations can be suggested to have impacted the native soil bacteria, although H_2_O_2_ used in a similar manner did not totally abolish the microbial population in contaminated soil in an earlier pilot scale test (Simpanen et al. 2016). We ensured the presence of a degradation-active microbiome by using an inoculum, i.e., adding soil that had recently been successfully remediated by biostimulation.

**Fig. 8:**
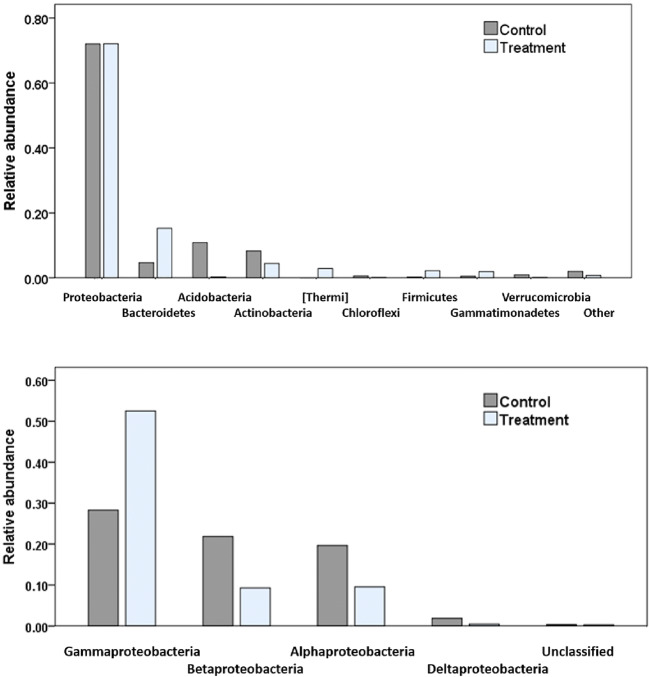
Bacterial relative abundance within phylum (**a**) and within proteobacteria in the two soils used for the inoculum (**b**) (> 1%)

In the two soils used, the differences in the final oil concentrations appeared to be related to the increase in relative abundance of bacteroidetes and also of gammaproteobacteria in relation to betaprotebacteria and alpahaprotebacteria within the proteobacteria phylum (Appendix 1 Fig. 8a&b). Both changes have been associated with bioremediation success for oil hydrocarbons (Siles and Margesin 2018). Gammaproteobacteria also constitute the dominant phylotype associated with both the presence and successful remediation of PAHs, especially in low nutrient environments (Parajuli et al. 2017; Roslund et al. 2019; Viñas et al. 2005). The two major families with heightened relative abundance within gammaproteobacteria were *Pseudomonadaceae* and *Xanthomonadaceae* (Appendix Table 4). Many *Pseudomonas* strains are known to utilize a multitude of aliphatic, aromatic and polyaromatic compounds of ecotoxicological concern in diverse nutrient conditions (Palleroni et al. 2010). Also, several genera within *Xanthomonadaceae* are known to be involved with oil hydrocarbon degradation (Chang and Zylstra 2010).

The necessity of bioaugmentation could not, however, be verified as the biological state of the soil was not tested prior or after the augmentation. A reduction in the average oil concentration down to ca. 10% of the initial level was achieved within the first 4 months of biostimulation, whereas only the first 10 cm under the tank jacket was affected. At this depth, the midrange fractions now represented less than 50% of the total hydrocarbons. Alkanes lighter than C21 are known to be biodegraded faster than the heavier fraction and changes in mid-to-heavy range ratio are therefore often used to approximate biodegradation progress (Kostka et al. 2011; Prince 2010; Rodriguez-Blanco et al. 2010). The mobilization of contaminant both within the soil and in the aqueous phase during chemical oxidation can be suggested to have been beneficial for subsequent biodegradation. The latter mechanism has been demonstrated by Talvenmäki et al. (2019), whereas the total effect was now likely negatively affected by the clayey soil type. Regardless of the mobilization mechanism, the contaminant was thus transported to a location with enhanced oxygen and nutrient availability.

Very little or no effect was observed in the time frame of 4 to 10 months, either in average concentrations of relative abundance of midrange fractions. Possible reasons for this were the low availability of hydrocarbons, or insufficient aeration between injections. A strong ammonia odor was also observed thorough the period, suggesting loss of nitrogen through volatilization when using ammonium fertilizers, resulting from increase in pH which may have also inhibited biodegradation.

When meat and bone meal (MBM) was introduced as the alternative nitrogen source, degradation was not visibly affected during the following 6-month period. The soil becoming increasingly water-saturated may have also negatively affected aerobic biodegradation. The addition of low concentrations of H_2_O_2_ as the oxygen source during this stage may have been insufficient. Peroxide is consumed by organic material, in this case possibly the MBM, before diffusion into deeper soil layers.

During the last bioremediation step, hydrocarbon and oxygen availability were the two factors targeted with the CD and CaO_2_ amendments. CaO_2_ has been successfully utilized as an oxygen releasing additive in aquatic applications (Nykänen et al. 2012), whereas degradation of the MBM is an oxygen demanding process potentially exceeding the availability from the slow release compound. Also, the effect of CD on solubility of oils has mostly been studied with circulated water phases, and can be expected to decrease with standing water bodies, such as the one found at the site during the later stages of the treatment (Simpanen et al. 2016). Regardless of these potential factors, the remediation effect was found to have reached the bottom of the permeable layer within 12 months and the total contaminant reduction was now 98% of the initial value. Similarly to what was observed during the early months of the biostimulation, the relative abundance of compounds in the C10–C21 range were more heavily affected, dropping to below 40% of the total hydrocarbon concentration in all samples (Table 3). When the effect of poor bioavailability was removed, the slow release compounds for oxygen and nutrients were hence found efficient to support continuous biological degradation, suggesting that bioavailability had been the primary bottleneck.

At the closure of the monitoring period, the C10–C40 concentration in the aqueous phase was 0.67 mg/L. The aqueous phase in the tank could also be removed easily, which was done during soil sampling. Due to the slow infiltration rate, it was suggested that the dissolved contaminant was likely to stay within the vicinity of the tank rather than be transported elsewhere. In this case, the tank conditions provided high enough average temperatures even during the cold season (approx. 0–10 °C), and hence the water was left in the tank. Also, due to the added slow release compounds for oxygen and nutrients, the remediation effect would continue even without active treatment interventions.

In the investigations performed by the contractor, the average C10–C40 concentration at a depth of 2–3 m was approximately 400 mg/kg, with only two out of six samples showing concentrations above 20 mg/kg (2070 mg/kg dw in B5; 149 mg/kg dw in B1, Fig. 3a). In composite samples at three different spots, at depth 2–3 m, 65% of the total concentrations consisted of aliphatic compounds in the C16–C35 range and 19% in the C12–C16 range. These results indicate that local hot spots could still be detected in the soil, and average values would therefore be largely affected by the number of hot spots encountered during sampling. It can also be suggested that due to the low permeability of the soil, the actions performed in the immediate tank area had not affected the entire outer perimeter of the contaminated zone. These soil samples were not water-saturated and because of this, it appears unlikely that the hot spot was caused by contaminant mobilization through preferential flow paths with higher permeability relative to the surrounding soil mass.

According to the verdict of the local authority, the Häme Centre for Economic Development, Transport and the Environment, based on the reports and the risk assessment, the site was no longer found to pose environmental or health risks requiring further treatment.

## Conclusion

The sequential in situ treatment of the site resulted in a successful outcome in a limited zone, this result being achieved with the joint effect of the different approaches. The treatment itself affected the site conditions and therefore, also the requirements for the subsequent treatment steps. Laboratory-scale tests were crucial, both in directing the choices of additives at the site, and also in demonstrating the associated risks and benefits to the environmental authorities. The total time for the treatment was considered needlessly long as periods of dormancy could be observed. If, however, slower progress is considered acceptable, the cautionary principle may be applicable, especially when the methods are known to potentially increase the risk level. In this investigation, these ineffective periods could also be considered as control periods during which vital reference data was collected, as similar sites with identical soil type and structure were not available for the study. With accumulating experience on how to combine different in situ techniques and in what order to use them most efficiently, future in situ remediation undertakings can be performed with shorter lag periods, reaching a satisfactory result in a significantly shorter treatment time.

## Data Availability

The authors confirm that the data supporting the findings of this study are available within the article.

## Appendix

**Table 4:** Relative abundance in Class and Order and Family and Genus levels in the two soils used for the inoculum

Class			Order		
	Control	Treatment		Control	Treatment
Gammaproteobacteria	0.283	0.525	Xanthomonadales	0.126	0.323
Betaproteobacteria	0.219	0.093	Sphingomonadales	0.108	0.026
Alphaproteobacteria	0.196	0.095	Alteromonadales	0.102	0.001
Actinobacteria	0.078	0.043	Actinomycetales	0.078	0.043
Holophagae	0.071	0.001	Burkholderiales	0.075	0.084
Flavobacteriia	0.003	0.051	Holophagales	0.070	0.001
Deltaproteobacteria	0.019	0.005	Rhizobiales	0.031	0.023
[Saprospirae]	0.015	0.040	Caulobacterales	0.023	0.042
iii1-8	0.012	0.000	Clostridiales	0.001	0.009
Acidobacteriia	0.010	0.000	Rhodospirillales	0.021	0.001
Gemmatimonadetes	0.004	0.017	PYR10d3	0.021	0.000
Bacteroidia	9.91E-05	0.036	Pseudomonadales	0.017	0.195
[Chloracidobacteria]	0.006	0.000	Sphingobacteriales	0.002	0.017
[Spartobacteria]	0.005	0.001	[Saprospirales]	0.015	0.04
Acidobacteria-6	0.005	0.000	DS-18	0.011	0.000
Sphingobacteriia	0.002	0.017	Acidobacteriales	0.010	0.000
Bacilli	0.002	0.013	Flavobacteriales	0.003	0.051
Others	0.071	0.064	Myxococcales	0.008	0.002
			Rhodobacterales	0.007	0.000
			Ellin6067	0.007	0.000
			RB41	0.005	0.000
			[Chthoniobacterales]	0.005	0.001
			Bacteroidales	9.91E-05	0.036
			Others	0.254	0.106
Family			Genus		
	Control	Treatment		Control	Treatment
Sphingomonadaceae	0.103	0.026	Arenimonas	0.010	0.000
Alteromonadaceae	0.101	0.001	B-42	0	0.029
Xanthomonadaceae	0.071	0.321	Caulobacter	0.013	0.003
Holophagaceae	0.070	0.001	Flavihumibacter	9.91E-05	0.006
Microbacteriaceae	0.062	0.023	Gelidibacter	0	0.027
Sinobacteraceae	0.049	0.001	Geothrix	0.070	0.001
Comamonadaceae	0.046	0.046	HB2-32-21	0.101	0.001
Oxalobacteraceae	0.026	0.002	Kaistobacter	0.005	0.000
Caulobacteraceae	0.023	0.042	Kaistobacter	0.007	0
Chitinophagaceae	0.0145	0.040	Kaistobacter	0.006	0
Moraxellaceae	0.013	0.002	Lysobacter	0	0.091
Acetobacteraceae	0.011	0.000	Lysobacter	0	0.005
Rhodospirillaceae	0.009	0.001	Novosphingobium	0.015	0
Bradyrhizobiaceae	0.007	0.001	Perlucidibaca	0.012	0.001
Rhodobacteraceae	0.007	0.000	Petrimonas	0	0.011
Hyphomicrobiaceae	0.007	0.002	Pseudomonas	0.003	0.015
Koribacteraceae	0.006	0.000	Pseudoxanthomonas	0.005	0.005
[Chthoniobacteraceae]	0.005	0.001	Rhodanobacter	0.001	0.080
Pseudomonadaceae	0.004	0.192	Roseococcus	0.007	0.000
Flavobacteriaceae	0.003	0.043	Sediminibacterium	0.013	0
Alcaligenaceae	0.001	0.035	Serpens	0	0.055
Porphyromonadaceae	0	0.035	Sinobacter	0.008	0
Trueperaceae	0	0.029	Sinobacter	0.006	0
Sphingobacteriaceae	0.002	0.017	Sinobacter	0.008	0
Phyllobacteriaceae	0.001	0.006	Sphingomonas	0.010	0.001
[Weeksellaceae]	0	0.005	Thermomonas	0	0.016
Clostridiaceae	0	0.005	Others	0.700	0.652
Others	0.355	0.121			
